# Dr Bridger

**Published:** 1825-01-01

**Authors:** 


### DR. BRIDGER.

Lately died, after a severe and protracted illness, at his lodgings in Sloane Street, Dr. Wm. Bridger, Surgeon in the Honorable East India Company's Naval Service. Dr. Bridger was a gentleman of remarkably amiable disposition, and pleasing manners. His illness began three or four years ago, as an affection of the mucous membrane of the lungs; for the relief of which he tried the milder skies of Southern Europe, but without benefit. The disease gained the parenchymatous structure of the lungs, and latterly, assumed the fatal form of phthisis. He was attended by Dr. Chambers and Dr. Johnson, who endeavoured to smooth the dreary path to that bourne whence no traveller returns!

